# Antibiotic Resistance and Virulence Profiles of *Escherichia coli* Strains Isolated from Wild Birds in Poland

**DOI:** 10.3390/pathogens10081059

**Published:** 2021-08-20

**Authors:** Anna Nowaczek, Marta Dec, Dagmara Stępień-Pyśniak, Renata Urban-Chmiel, Agnieszka Marek, Paweł Różański

**Affiliations:** 1 Department of Veterinary Prevention and Avian Diseases, Faculty of Veterinary Medicine, University of Life Sciences in Lublin, Akademicka 12, 20-033 Lublin, Poland; anna.nowaczek@up.lublin.pl (A.N.); dagmara.stepien@up.lublin.pl (D.S.-P.); renata.urban@up.lublin.pl (R.U.-C.); agnieszka.marek@up.lublin.pl (A.M.); 2 Department of Animal Hygiene and Environmental Hazards, Faculty of Animal Sciences and Bioeconomy, University of Life Sciences in Lublin, Akademicka 13, 20-950 Lublin, Poland; pawel.rozanski@up.lublin.pl

**Keywords:** *Escherichia coli*, APEC, wild birds, virulence genes, resistance genes, antibiotic resistance, MIC

## Abstract

Wild animals are increasingly reported as carriers of antibiotic-resistant and pathogenic bacteria including *Enterobacteriaceae*. However, the role of free-living birds as reservoirs for potentially dangerous microbes is not yet thoroughly understood. In our work, we examined *Escherichia coli* strains from wild birds in Poland in relation to their antimicrobial agents susceptibility, virulence and phylogenetic affiliation. Identification of *E. coli* was performed using MALDI-TOF mass spectrometry. The antibiotic susceptibility of the isolates was determined by the broth microdilution method, and resistance and virulence genes were detected by PCR. *E. coli* bacteria were isolated from 32 of 34 samples. The strains were most often classified into phylogenetic groups B1 (50%) and A (25%). Resistance to tetracycline (50%), ciprofloxacin (46.8%), gentamicin (34.3%) and ampicillin (28.1%) was most frequently reported, and as many as 31.2% of *E. coli* isolates exhibited a multidrug resistance phenotype. Among resistance genes, *sul2* (31.2% of isolates) and *bla_TEM_* (28.1%) were identified most frequently, while *irp-2* (31.2%) and *ompT* (28.1%) were the most common virulence-associated genes. Five strains were included in the APEC group. The study indicates that wild birds can be carriers of potentially dangerous *E. coli* strains and vectors for the spread of resistant bacteria and resistance determinants in the environment.

## 1. Introduction

*Escherichia coli* (*E. coli*) is a common member of the natural intestinal microflora of humans and animals, including birds. However, in addition to non-pathogenic commensal strains, there are also pathogenic *E. coli* strains involved in many types of infections. Intestinal pathogenic *E. coli* (IPEC) bacteria are associated with infections of the gastrointestinal tract, while extraintestinal pathogenic *E. coli* (ExPEC) strains cause infections in extraintestinal anatomic sites. Several pathotypes can be distinguished among IPEC strains: enterotoxigenic *E. coli* (ETEC), enteropathogenic *E. coli* (EPEC), enterohaemorrhagic *E. coli* (EHEC), enteroinvasive *E. coli* (EIEC), enteroaggregative *E. coli* (EAEC), adherent invasive *E. coli* (AIEC), and diffusely adherent *E. coli* (DAEC) [1]. ExPEC strains include avian pathogenic *E. coli* (APEC), uropathogenic *E. coli* (UPEC), neonatal meningitis *E. coli* (NMEC), and sepsis-associated *E. coli* (SEPEC) [1,2]. Characteristic virulence factors, which play a decisive role in pathogenesis, have been defined for each of these pathotypes [3].

APEC strains are responsible for different extraintestinal diseases in birds, known as avian colibacillosis. In poultry, these infections can be local or systemic, and include acute coli-septicemia, fibrinopurulent polyserositis, aero-sacculitis, pericarditis, salpingitis, synovitis, omphalitis, yolk sac infection, swollen head syndrome, coli-granuloma, and cellulitis [4]. Due to their high incidence and mortality, these diseases cause huge economic losses in the poultry sector around the world.

APEC strains produce a variety of virulence factors facilitating host tissue colonization, including adhesins (encoded by *papC* and *tsh*), iron uptake systems (*iutA*, *irp2*, *sit* and *iroN*), serum resistance (*iss*, *ompT* and *kpsII*), toxins (*vat*), and others (*cvi/cva* and *etsB*) [5]. Many genes coding for these virulence factors are often clustered together on chromosomes and plasmids and can be transmitted by horizontal gene transfer [6]. Moreover, APEC and other ExPEC strains that cause infections in humans, are quite closely phylogenetically related and share some of the same virulence genes. Therefore, APEC strains may hypothetically have zoonotic potential and pose a health risk to humans [7].

In recent years, a significant increase in antibiotic resistance has been noted not only among pathogenic strains, but also among commensal *E. coli*. Antibiotic-resistant *E. coli* strains are currently found in a variety of environments, and their spread is promoted by the extreme genomic plasticity of these bacteria [8]. A report from the European Antimicrobial Resistance Surveillance Network (EARS-Net) from 2020 shows that more than half (57.1%) of the *E. coli* strains from humans reported in 2019 showed resistance to at least one of the groups of antibiotics under surveillance. Resistance to penicillin was most commonly reported (57.1%), followed by fluoroquinolones (23.8%), third-generation cephalosporins (15.1%), and aminoglycosides (10.8%) [9]. A high percentage of antibiotic-resistant *E. coli* isolates is also noted in farm animals, especially poultry. In Poland, *E. coli* strains from broiler chickens are most commonly found to be resistant to penicillin, fluor-quinolones and tetracyclines, and antibiotic resistance applies both to isolates from non-clinical birds and to cases of colibacillosis [10].

The occurrence of resistant *E. coli* bacteria in wild birds, including multidrug resistant strains, has been demonstrated by several authors conducting research in various countries around the world, including Poland [11,12,13,14,15]. They can also be a reservoir of APEC, as well as *E. coli* strains pathogenic to humans, including *E. coli* serotype H7:O157 [16,17]. Due to their migratory lifestyle, free-living birds can be contributing vectors for the spread of potentially dangerous *E. coli* strains between ecosystems.

Information on the characteristics of *E. coli* strains in free-living birds in Poland is limited. Therefore, we have conducted a study aimed at assessing the occurrence of antibiotic-resistant and virulent *E. coli* from wild birds inhabiting suburban areas of south-eastern Poland. Referring to this fact, the intention of the study was to determine, whether wild birds are carriers and transmitters of dangerous *Enterobacteriaceae* strains that, when spreading in the environment, may pose a threat to poultry farms.

## 2. Results

### 2.1. Identification of E. coli

*E. coli* bacteria were isolated from 32 of 34 samples taken from wild birds. Log(score) values obtained in MALDI-TOF mass spectrometry were higher than 2300 for all these isolates, which indicates a high probability of correct identification to the species level (Table 1). The largest number of *E. coli* isolates (*n* = 13) was obtained from mallards (*Anas platyrhynchos*), and the rest from white-tailed eagle (*Haliaeetus albicilla*) (*n* = 2), common buzzard (*Buteo buteo*) (*n* = 2), Eurasian sparrow hawk (*Accipiter nisus*) (*n* = 2), Eurasian tawny owl (*Stix aluco*) (*n* = 2), mute swan (*Cygnus olor*) (*n* = 1), little bittern (*Ixobrychus minutus*) (*n* = 1), little owl (*Athene noctua*) (*n* = 1), short-eared owl (*Asio flammeus*) (*n* = 1), great spotted woodpecker (*Dendrocopos major*) (*n* = 1), lesser spotted woodpecker (*Dendrocopos minor*) (*n* = 1), European green woodpecker (*Picus viridis*) (*n* = 1), bohemian waxwing (*Bombycilla garrulus*) (*n* = 1), western capercaillie (*Tetrao urogallus*) (*n* = 1), grey heron (*Ardea cinerea*) (*n* = 1), and Eurasian golden oriole (*Oriolus oriolus*) (*n* = 1) (Table 1). Two birds from which we did not isolate *E. coli* belonged to the species peregrine falcon (*Falco peregrinus*). From cloacal swabs from these birds, bacterial growth on MacConkey agar was obtained; however, these strains were identified by MALDI-TOF MS as *Escherichia* *albertii* (data not shown).

### 2.2. Antibiotic Resistance of E. coli

Multidrug-resistant (MDR) bacteria show non-susceptibility to at least one agent in three or more antimicrobial categories [18]. Analysis of resistance to antimicrobial agents based on MICs showed that 31.2% (*n* = 10) of the *E. coli* strains were resistant to three or more groups of antibiotic and 28.1% (*n* = 9) were resistant to two groups of antibiotic (Table 1). Only one *E. coli* isolate (e48) was found to be susceptible to all antimicrobials tested. As many as, 50% of isolates (*n* = 16) were resistant to tetracycline and 10 strains showed intermediate susceptibility to this antibiotic. Resistance to ciprofloxacin was demonstrated in 46.8% (*n* = 15) of isolates. Among aminoglycosides, 34.3% (*n* = 11) of isolates were resistant to gentamicin, and 40.6% (*n* = 13) showed intermediate susceptibility to this antimicrobial agent, while 18.7% (*n* = 6) of *E. coli* strains showed resistance to kanamycin. Lower incidence of resistance was found for trimethoprim/sulfamethoxazole (34.3%, *n* = 11) and ampicillin (28.1%, *n* = 9). Resistance to chloramphenicol was noted sporadically, with only two (6.25%) resistant isolates and one with intermediate susceptibility (3.1%) (Table 2). MDR *E. coli* isolates came from mallard (*Anas platyrhynchos*) (*n* = 6), Eurasian golden oriole (*Oriolus oriolus*) (*n* = 1), Eurasian sparrow hawk (*Accipiter nisus*) (*n* = 1), common buzzard (*Buteo buteo*) (*n* = 1) and little bittern (*Ixobrychus minutus*) (*n* = 1). Among the MDR *E. coli* strains, the most common resistance profile was tetracycline–trimethoprim/sulfamethoxazole–ampicillin (Table 1).

### 2.3. Detection of Resistance and Virulence Genes

All isolates showing resistance to ampicillin (*n* = 9) contained the *bla_TEM_* gene (encoding β-lactamase TEM capable of inactivating penicillin, including ampicillin), and the *aphA1* gene (coding for aminoglycoside 3′-phosphotransferase) was found in all kanamycin-resistant *E. coli* strains (*n* = 6). The *sul2* gene (encoding the mutant dihydropteroate synthase enzyme that does not bind sulfonamides) was detected in 10 of 11 *E. coli* phenotypically resistant to trimethoprim/sulfamethoxazole, the *sul3* gene was detected in three strains, and one strain resistant to these two antibiotics additionally had the *dhfrI* gene (mediating trimethoprim resistance). The *tetA* gene was found in four of 16 isolates showing resistance to tetracycline. Regarding streptomycin resistance genes, which belong to aminoglycoside group, *aadA* was found in two of all examination strains and gene *strA/strB* was also found in two strains. None of the *E. coli* isolates contained the *tetB*, *aac(3)-IV*, *aac(3)-II*, *aphA2*, *qnr*, *catI* or *sul1* gene (Table 1).

Nine of the 24 virulence genes were detected in the *E. coli* isolates. The most commonly detected were *irp-2* (found in 31.2% of isolates, *n* = 10,) *ompT* (28.1%, *n* = 9), *iutA* and *iss* (21.8%, *n* = 7 each). Other virulence genes typical of APEC strains, i.e., *cva/cvi*, *iucD*, *pap-C* and *tsh*, were detected less frequently. Furthermore, the *astA* gene characteristic of enteroaggregative *E. coli* strains (EAEC) was recorded in four isolates. Nine (28.1%) *E. coli* strains contained multiple virulence genes (at least three), but none of the virulence genes were detected in 37.5% (*n* = 12) strains (Table 1).

Five strains that contained at least five virulence genes were classified as APEC. Three strains (from mallard) showed coexistence with five APEC-specific virulence genes (*ompT* + *iutA* + *iucD* + *iss* + *cva/cvi*), one isolate (from Eurasian sparrow hawk) contained six virulence genes (*ompT* + *iutA* + *iucD* + *irp-2* + *iss* + *cva/cvi*), and one strain (from little bittern) had six genes (*ompT* + *iutA* + *iucD* + *irp-2* + *iss* + *cva/cvi* + *astA*) characteristic of APEC as well as *astA*. 

### 2.4. Determination of E. coli Phylogenetic Groups

**Figure 1:**
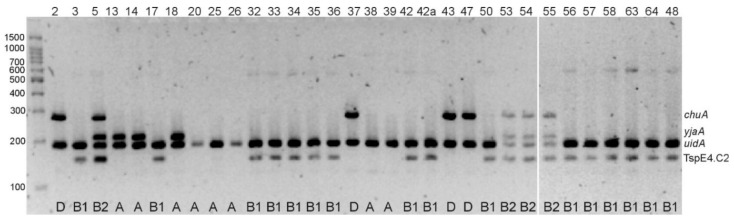
Multiplex PCR patterns for *E. coli* phylogenetic groups.

Phylogenetic groups of *E. coli* were determined based on the electrophoretic profiles of multiplex PCR amplicons (*yja*, TspE4.C2, *chuA*, *svg* and *uidA*). Half (50%) of the isolates (*n* = 16) belonged to group B1, 25% of isolates (*n* = 8) were assigned to group A, 12.5% (*n* = 4) to B2 and 12.5% (*n* = 4) to group D. None of the isolates belonged to group B2_1_ (all isolates were *svg*-negative) (Figure 1). 

Most of the MDR strains belonged to the type A phylogenetic group (seven of 10 MDR strains), two to group B1 and one to group D. Strains containing virulence factors (*n* = 21.7%) were assigned to various phylogenetic groups, including group A (*n* = 7), B1 (*n* = 5), B2 (*n* = 4) and D (*n* = 4). As many as 10 of 12 isolates in which no virulence gene was detected represented phylogenetic group B1. In addition, seven isolates in group B1 were negative for both virulence and resistance genes.

The incidence of virulence genes *ompT*, *iutA*, and *iucD* in the phylogenetic group B1 is significantly lower than in A, B2 and D. The observed relationship is at an average level as indicated by the analysis of the contingency coefficient (0.52, 0.47, 0.48, respectively). For these three genes, a significant correlation was also observed in the decrease in virulence with the increase in the number of observations in the B1 = phylogenetic group, which confirms the strength of the relationship observed. Taking into account all the virulence genes tested, the B1 phylogenetic group was significantly less virulent (*p* = 0.009) than the others (contingency coefficient 0.55).

## 3. Discussion

So far, only a few studies have been carried out on antibiotic resistance and the presence of virulence genes in *E. coli* bacteria from free-living birds in Poland. Antibiotic-resistant *E. coli* strains, including MDR, are widely isolated from humans [19], farm animals [20,21] and increasingly from wildlife [22]. Our study has shown that resistant *E. coli* strains, including MDR, are spread among wild birds. The frequency of resistance is much higher than that observed for *E. coli* originating in water birds from the Baltic Sea coast in Poland [14] and in birds living in other parts of Europe [11,12,23]. 

The high frequency of tetracycline resistance (50%) in the strains tested in our study is consistent with the results of Pinto et al. [13] and Radhauani et al. [12], who found that over 70% of *E. coli* from Portuguese wild birds showed a tetracycline-resistant phenotype. It should be emphasized, however, that several other authors noted less than 22% tetracycline-resistant *E. coli* from free-living birds [11,23,24,25,26]. In *E. coli* isolates from poultry, the frequency of resistance to tetracycline is over 70% [27,28,29]. The *tet(A)* gene found in several phenotypically resistant strains is often detected in Enterobacteriaceae from poultry [30] and was recorded in *E. coli* isolates from wild birds of the Azores Archipelago [31].

In the case of ampicillin, the resistance rate in *E. coli* strains was 28.1%, and the results were similar to those reported by other authors [11,24,26], who identified 18.1–19.5% of isolates as ampicillin-resistant. In poultry, significantly higher resistance to ampicillin (≥63%) is generally found [27,28]. Phenotypic resistance correlated with the presence of the *bla_TEM_* gene, that was also previously found in ampicillin-resistant *E. coli* from buzzards (*Buteo buteo*) in Portugal and wild birds in Poland [12,14].

The percentage of ciprofloxacin-resistant isolates reported in our study (nearly 50%) is consistent with the results of Radhauani et al. [12], who found a resistance rate of 50% in *E. coli* isolates from buzzards. Other authors, however, note a much lower frequency of fluoroquinolone resistance in *E. coli* from wild birds, ranging from 3% to 22% [24,25], and some do not report resistance to this group of antimicrobial agents at all [15,23]. In *E. coli* from broiler chickens, the percentage of isolates resistant to ciprofloxacin is usually high, above 50% [27,28].

An interesting finding was the high percentage of gentamicin-resistant strains confirmed in our study (75%), which significantly differs from the results of other research projects. Several authors [15,24,26] observed fewer than 1% gentamicin-resistant strains among *E. coli* isolates from wild birds. To rule out any error in determining the gentamicin MIC value, we additionally used the disc diffusion method (data not shown). Despite the high percentage of gentamicin-resistant strains, the resistance genes that usually determine resistance to gentamicin in *Enterobacteriaceae*, i.e., *aac(3)-II* and *aac(3)-IV* (coding for aminoglycoside acetyltransferase), were not detected. It is therefore possible that resistance to gentamicin in the strains is the result of the production of other aminoglycoside-modifying genes (e.g., *ant-2*) or other resistance mechanisms [32].

The percentage of trimethoprim/sulfamethoxazole-resistant isolates recorded in our study (over 35%) is similar to the results of Radhouani et al. [12], who classified 22% of *E. coli* isolates from common buzzards in Portugal as phenotypically resistant. A much lower frequency of trimethoprim/sulfamethoxazole-resistant *E. coli* strains, of less than 5%, was observed in isolates from European starlings (*Sturnus vulgaris*) [15]. In line with other studies, we also note the common presence of the *sul2* gene in trimethoprim/sulfamethoxazole-resistant *E. coli* isolates [15].

A high percentage of antibiotic-resistant strains found in wild birds in this study may be the result of acquisition of resistant strains from human sources, farms, or contaminated river water [33]. Wastewater and outflows from fields fertilized with manure make their way into rivers, and fecal bacteria may thus be transported over long distances.

Virulence genes characteristic of APEC, as well as the *astA* gene, were detected in most of the *E. coli* isolates. The most frequently identified gene was *irp-2*, which encodes iron-repressible high-molecular weight protein 2 (IRP-2), involved in iron acquisition. Some authors show a high prevalence of this gene among EAEC strains, although the role of IRP-2 in EAEC pathogenesis in unclear [34]. The *irp-2* gene recorded in this study, as well as other genes suggested as predictors of the virulence of APEC strains, i.e., *ompT*, *iutA*, *iss*, *pap-C*, *cva/cvi* and *tsh*, are commonly found in pathogenic *E. coli* strains from poultry [35], but have also been reported in *E. coli* from wild birds [36]. Kuczkowski et al. [36] demonstrated the frequent occurrence of the *irp-2* and *astA* genes (in 11.6% of isolates), while the *iss*, *iucD*, *tsh*, *pap-C* and *vat* genes were rarely found in *E. coli* isolates from wild water birds in Poland and the Netherlands. Borges et al. [16] showed that 30.6% of *E. coli* isolates taken from wild birds in Brazil were positive for at least one virulence gene, the most prevalent being *iss*, followed by *sitA*, *traT*, *ompT*, *fyuA* and *irp2*. Other virulence-associated genes, including *iutA*, *iucC*, *iucD*, *tsh* and *cvaC*, were detected sporadically. The *astA* gene found in several isolates is responsible for the production of heat-stable enterotoxin 1 (EAST1) characteristic of diarrheagenic EAEC strains [37]. The presence of the *astA* gene has also been confirmed in ExPEC strains derived from both humans (UPEC) and poultry (APEC) [36,38]. However, ExPEC isolates may also contain an *astA* pseudogene that has not been reported in diarrheagenic *E. coli* strains [38].

The dominance of *E. coli* group B1 isolates in this study is consistent with the results of other authors analyzing *E. coli* from wild birds [36,39]. Commensal *E. coli* with no pathogenic features which inhabit the gastrointestinal tract most often represent group A or B1, while ExPEC strains (including APEC) are usually assigned to phylogenetic groups B2 and D [2]. The *E. coli* strains used in these studies were not tested for pathogenicity, but we have noted a significantly lower incidence of virulence genes in isolates of the phylogenetic group B1. Four out of five strains qualified as APEC represented phylogenetic group A, and one strain represented group B1.

## 4. Materials and Methods

### 4.1. Isolation of Escherichia coli Strains

The material for the study was fresh feces from 34 wild birds of different species in mainly habiting suburban areas of south-eastern Poland. One sample per bird was collected within 1–2 h after the birds had been transported to the Wild Bird Rehabilitation Centre at the Department and Clinic of Animal Surgery, Faculty of Veterinary Medicine, University of Life Sciences in Lublin. The material was collected between May and October 2017. Samples were recovered from 17 wild bird species: mallard (Anas platyrhynchos, *n* = 13), white-tailed eagle (*Haliaeetus albicilla*, *n* = 2), common buzzard (*Buteo buteo*, *n* = 2), Eurasian sparrow hawk (*Accipiter nisus*, *n* = 2), peregrine falcon (*Falco peregrines*, *n* = 2), Eurasian tawny owl (*Stix aluco*, *n* = 2), mute swan (*Cygnus olor*, *n* = 1), little bittern (*Ixobrychus minutes*, *n* = 1), little owl (*Athene noctua*, *n* = 1), short-eared owl (*Asio flammeus*, *n* = 1), great spotted woodpecker (*Dendrocopos major*, *n* = 1), lesser spotted woodpecker (*Dendrocopos minor*, *n* = 1), European green woodpecker (*Picus viridis*, *n* = 1), bohemian waxwing (*Bombycilla garrulous*, *n* = 1), western capercaillie (*Tetrao urogallus*, *n* = 1), grey heron (*Ardea cinerea*, *n* = 1) and Eurasian golden oriole (*Oriolus oriolus*, *n* = 1). Feces was suspended in buffered peptone water (Oxoid Ltd., Basingstoke, UK) and subsequently, a loopful from each suspension was inoculated directly onto MacConkey agar (Oxoid Ltd., Basingstoke, UK) plates and incubated at 37 °C for 24 h under aerobic conditions. Single pink colonies were harvested, cultured on BHI (Brain Heart Infusion) broth (Oxoid Ltd., Basingstoke, UK) and pure cultures supplemented with 20% glycerol were stored at −80 °C for further analysis.

### 4.2. Identification of E. coli

The species of the isolates were confirmed by MALDI-TOF mass spectrometry (UltrafleXtreme MALDI-TOF, Bruker Daltonics, Hamburg, Germany) using a standard ethanol/formic acid extraction sample preparation procedure [40]. The mass spectra obtained from each isolate were processed with the MALDI Biotyper 3.0 software package (Bruker Daltonics, Hamburg, Germany), and the results were shown as the top 10 identification matches along with confidence scores ranging from 0.000 to 3.000, according to the manufacturer’s criteria (www.bruker.com; accessed on: 19 September 2021).

### 4.3. Antibiotic Susceptibility Testing

Antibiotic profiles of *E. coli* strains were based on determination of the minimum inhibitory concentration (MIC) of the antibiotic defined by serial microdilution in Mueller-Hinton broth (Oxoid Ltd., Basingstoke, UK) on a 96-well flat-bottomed microplate (Medlab, Raszyn, Poland), according to standards developed by the Clinical and Laboratory Standards Institute [41].

The antimicrobials tested were ciprofloxacin (0.125–128 μg/mL), ampicillin (0.25–256 μg/mL), gentamicin (0.125–256 μg/mL), tetracycline (0.5–256 μg/mL), chloramphenicol (0.5–256 μg/mL), kanamycin (0.25–256 μg/mL), and trimethoprim/sulfamethoxazole (0.25–64 μg/mL and 4.75–1216 μg/mL, respectively, mixed at a 1:19 ratio). All dry powder antibiotics were purchased from Roth, Zielona Góra, Poland), except trimethoprim and sulfamethoxazole, which were obtained from Merck KGaA (Darmstadt, Germany), and ciprofloxacin, obtained from Honeywell-Fluka (Bucharest, Romania).

The *E. coli* colonies grown on Columbia agar with 5% defibrinated horse blood were suspended in 0.85% NaCl solution to obtain a density corresponding to 0.5 on the McFarland scale. Microdilution plates were inoculated with 50 µL of a 1:100-diluted (in Mueller-Hinton broth) inoculum and 50 µL of the appropriate antibiotic concentration (stock solutions were previously dissolved in Mueller-Hinton broth). Plates were incubated at 37 °C for 24 h under aerobic conditions [42]. An *Escherichia coli* ATCC 25922 reference strain was used as quality control. MIC breakpoint was defined as the lowest concentration of the substance at which no growth of the bacterial strains could be seen. Isolates were classified as susceptible, intermediate and resistant according to the threshold breakpoint proposed by the Clinical and Laboratory Standards Institute [41].

### 4.4. Detection of Resistance Genes

Genomic DNA from *E. coli* isolates was isolated using a Gene MATRIX Bacterial & Yeast Genomic DNA Purification Kit (Eurx, Gdańsk, Poland) following the manufacturer’s instructions. The presence of genes conferring resistance to tetracyclines—*tet(A)* and *tet(B)*; aminoglycosides—*aphA1*, *aphA2*, *aac(3)-II* and *aac(3)-IV*; sulfonamides—*sul1*, *sul2* and *sul3*; β-lactams—*bla_TEM_*; fluoroquinolones—*qnr*; trimethoprim—*dhfrI*; and chloramphenicol—*catI* and *cm1A*—were determined by PCR using the primers presented in Table A1. The reaction mixture used in the PCR assay contained 2.5 μL of 10× concentrated reaction buffer containing 25 mM MgCl_2_, 1 μL 25mM dNTPs mix, 1U AllegroTaq polymerase (Novazym, Poznań, Poland), 1 μL of each of the primers (10 μM) and 1 μL of the tested *E. coli* DNA in a total volume of 25 μL for each sample. Amplification reactions were performed in a thermocycler (Eppendorf Mastercycler Gradient, Westbury, NY, USA) using the following program: initial denaturation—1 cycle at 94 °C for 5 min, 30 cycles of 40 s at 94 °C, 40 s at 50–66 °C (according to the annealing temperature for the individual primers; Table A1), and 75 s at 72 °C, followed by 8 min of final extension at 72 °C. The PCR products in a volume of 8 μL were separated by electrophoresis (100 V) on a 1.5% agarose gel in 1 × TBE (Tris-borate-EDTA) buffer and visualized by SimplySafe staining (Eurx, Poland). A 100–1000 bp molecular standard (Blirt, Gdańsk, Poland) was used to determine the size of the amplification products, using Quantity One software (BioRad, Hercules, CA, USA).

### 4.5. Detection of Virulence Genes

Uni-plex or multiplex PCR, using gene-specific primers (Table A2 and Table A3), was used to detect the presence of 24 genes associated with virulence in *E*. *coli* strains. Three *E. coli* strains containing virulence genes were used as positive controls *(stx1*- and *stx2*-; *eaeA*- and *hlyA*-; *astA*-, *escV*- and *eaeA*-positive strains). Based on criteria described by De Carli et al. [43], isolates containing at least five virulence genes were considered APEC strains and isolates containing fewer than five virulence genes were considered avian non-pathogenic *Escherichia coli* (non-APEC) strains.

### 4.6. Determination of E. coli Phylogenetic Groups

To determine the phylogenetic groups of the *E. coli* isolates, five sets of primers for the genes *yja*, TspE4.C2, *chuA*, *svg* and *uidA* were used in a multiplex PCR, as previously described [44]. PCR products were separated by electrophoresis in 3% (*w*/*v*) high resolution agarose (Blirt, Gdańsk, Poland). The phylogenetic groups were determined based on the PCR gel pattern.

### 4.7. Statistical Analysis

In order to identify associations between phylogenetic groups of isolates and the presence of virulence-associated genes, the Chi-square independence test with Yates correction was used. The level of significance was set as *p* < 0.05. The statistical analysis was performed with the use of Statistica 13 data analysis software system, TIBCO Software Inc. 2017 (Palo Alto, CA, USA).

## 5. Conclusions

To sum up, the results of the study indicate that free-living birds can be a reservoir of *E. coli* strains containing both resistance and virulence genes, and due to their migratory life cycle they can contribute to the spread of resistant microbes between ecosystems. Given the large number of birds migrating in Europe each year, their contribution to the spread of drug-resistant and avian pathogenic strains of *E. coli* appears to be significant.

Monitoring the presence of antibiotic-resistant and pathogenic microorganisms in wild animals in various geographical areas makes it possible to assess the impact of the spread of resistance genes on humans and animals and enables the implementation of possible measures to control antibiotic resistance.

Further research is needed to clarify the high percentage of gentamicin-resistant *E. coli* strains not previously found in birds, and the mechanisms of resistance to this antibiotic.

## Figures and Tables

**Table 1:** Antibiotic resistance phenotypes, resistance genes, virulence genes and phylogenetic groups of all *E. coli* isolates.

Isolate	Species	MALDI-TOF MS Biotyper Log (Score)	Phylogenetic Group	Antibiotic Phenotype Pattern (Including Resistant and Intermediate Strains) ^a^	Resistance Genes	Virulence Genes
e2	White-tailed eagle (*Haliaeetus albicilla*)	*E. coli* 2.348	D	**T-CN-K**	*aphA1*	*astA*
e3	White-tailed eagle (*Haliaeetus albicilla*)	*E. coli* 2.305	B1	CN-**CIP**	-	-
e5	Mute swan (*Cygnus olor*)	*E. coli* 2.470	B2	T	-	*ompT*
e13	Eurasian golden oriole (*Oriolus oriolus*)	*E. coli* 2.442	A	**T-TR/S-AMP ^b^**	*tet(A)*, *sul2*, *blaTEM*, *strA/strB*	*astA*
e14	Eurasian sparrow hawk (*Accipiter nisus*)	*E. coli* 2.395	A	**T-TR/S-AMP ^b^**	*sul2, blaTEM*, *strA/strB*	*ompT*, *iutA*, *iucD*, *irp-2*, *iss*, *cva/cvi*
e17	Mallard (*Anas platyrhynchos*)	*E. coli* 2.316	B1	CN-**CIP**	-	-
e18	Mallard (*Anas platyrhynchos*)	*E. coli* 2.328	A	**T-TR/S-AMP-C ^b^**	*tet(A)*, *sul3*, *dhfrI*, *blaTEM, aadA*	*ompT*
e20	Mallard (*Anas platyrhynchos*)	*E. coli* 2.316	A	CN-**CIP-TR/S-AMP ^b^**	*sul2*, *blaTEM*	*ompT*, *iutA*, *iucD*, *iss*, *cva/cvi*
e25	Mallard (*Anas platyrhynchos*)	*E. coli* 2.378	A	**T**-CN-**CIP-TR/S-AMP ^b^**	*tet(A)*, *sul2*, *blaTEM*	*ompT*, *iutA*, *iucD*, *iss*, *cva/cvi*
e26	Mallard (*Anas platyrhynchos*)	*E. coli* 2.403	A	**CIP-TR/S-AMP ^b^**	*sul2*, *blaTEM*	*ompT*, *iutA*, *iucD*, *iss*, *cva/cvi*
e32	Eurasian tawny owl (*Stix aluco*)	*E. coli* 2.334	B1	**T**-CN	-	-
e33	Mallard (*Anas platyrhynchos*)	*E. coli* 2.603	B1	**T-CN-K**	*aphA1*	-
e34	Mallard (*Anas platyrhynchos*)	*E. coli* 2.344	B1	**T-CN-K**	*aphA1*	-
e35	Little bittern (*Ixobrychus minutus*)	*E. coli* 2.406	B1	**T-CN-CIP-TR/S-AMP-K-C ^b^**	*tet(A)*, *sul2*, *sul3*, *blaTEM*, *aphA1 aadA*	*astA*, *ompT*, *iutA*, *iucD*, *irp-2*, *iss*, *cva/cvi*
e36	Eurasian sparrow hawk (*Accipiter nisus*)	*E. coli* 2.370	B1	T-**CN-CIP**	-	-
e37	Mallard (*Anas platyrhynchos*)	*E. coli* 2.456	D	T-**CN**	-	*iss*
e38	Mallard (*Anas platyrhynchos*)	*E. coli* 2.304	A	**T-CN**	-	*astA*, *ompT*, *tsh*
e39	Mallard (*Anas platyrhynchos*)	*E. coli* 2.412	A	**T-CN-TR/S ^b^**	*sul2*	-
e42	Common buzzard (*Buteo buteo*)	*E. coli* 2.310	B1	**T-CN-CIP-TR/S-K-C ^b^**	*sul2*, *aphA1*	-
e42a	Common buzzard (*Buteo buteo*)	*E. coli* 2.345	B1	**T-CN-K**	*aphA1*, *sul3*	-
e43	Little owl (*Athene noctua*)	*E. coli* 2.402	D	CN-**TR/S-AMP**	*sul2*, *blaTEM*	*iutA*, *pap-C*, *irp-2*
e47	Mallard (*Anas platyrhynchos*)	*E. coli* 2. 376	D	**T-CN-CIP-TR/S-AMP ^b^**	*sul2*, *blaTEM*	*iutA*, *pap-C*, *irp-2*
e48	Mallard (*Anas platyrhynchos*)	*E. coli* 2.502	B1	-	-	-
e50	Mallard (*Anas platyrhynchos*)	*E. coli* 2.315	B1	**T**-CN-**CIP**	-	-
e53	Lesser spotted woodpecker (*Dendrocopos minor*)	*E. coli* 2.506	B2	T-CN	-	*irp-2*
e54	European green woodpecker (*Picus viridis*)	*E. coli* 2.317	B2	T-CN	-	*irp-2*
e55	Bohemian waxwing (*Bombycilla garrulus*)	*E. coli* 2.404	B2	T	-	*irp-2*
e56	Eurasian tawny owl (*Stix aluco*)	*E. coli* 2.348	B1	T-CN-**CIP**	-	*irp-2*
e57	Short-eared owl (*Asio flammeus*)	*E. coli* 2.372	B1	T-CN-**CIP**	-	-
e58	Great spotted woodpecker (*Dendrocopos major*)	*E. coli* 2.512	B1	**T**-CN-**CIP**	-	*irp-2*
e63	Western capercaillie (*Tetrao urogallus*)	*E. coli* 2.309	B1	T-CN-**CIP**	-	*irp-2*
e64	Grey heron (*Ardea cinerea*)	*E. coli* 2.324	B1	T-**CIP**	-	*ompT*, *iss*, *cva/cvi*

Legend: ^a^ bold and underlined symbols indicate resistance, non-bold, non-underlined symbols indicate intermediate susceptibility; T—tetracycline, C—gentamicin, K—kanamycin, CIP—ciprofloxacin, AMP—ampicillin, C—chloramphenicol, TR/S—trimethoprim/sulfamethoxazole, ^b^ MDR strain.

**Table 2:** Distribution of minimal inhibitory concentrations (MICs) of antibiotics among *Escherichia coli* isolates.

Antibiotic	Number of Isolates with MIC (µg/mL) of												Number of Resistant Isolates *n*/(%)
	≤0.125	0.25	0.5	1	2	4	8	16	32	64	128	≥256	
TE				1	1	4	10	13	2	1			16/(50%)
CN	1	2	1	1	3		13	9	2				11 (34.3%)
CIP	9	6	2			3	7	4			1		15/(46.8%)
AMP				4	10	6	3					9	9/(28.1%)
K			2	2			3	19		6			6/(18.7%)
C				8	18	2	1	1	1	1			2/(6.2%)
	**Number of Isolates with MIC (µg/mL) of**												
				≤0.25/4.75	0.5/9.5	1/19	2/38	4/76	8/152	16/304	32/608	≥64/1216	
TR/S					7	4	10	9	1	1			11/(34.3%)

Legend: dark grey: values for resistant strains; light gray: values for strains with intermediate susceptibility.

## Data Availability

All data generated or analysed during this study are included in this published article and its appendix information files.

## Appendix A

Table A1: Primer sequences for resistance genes and PCR conditions, Table A2: PCR primers used for detection of virulence genes, Table A3: PCR schemes used to detect virulence genes in *E. coli.*

**Table A1:** Primer sequences for resistance genes and PCR conditions.

Target Gene	Sequence (5′ → 3′)	Annealing Temperature (°C)	Size of Amplification Product (bp)	Reference
*aphA1*	F: ATG GGC TCG CGA TAA TGT C R: CTC ACC GAG GCA GTT CCA T	50	600	[45]
*aphA2*	F: GAA CAA GAT GGA TTG CAC GC R: GCT CTT CAG CAA TAT CAC GG	50	680	[45]
*sul1*	F: TTC GGC ATT CTG AAT CTC AC R: ATG ATC TAA CCC TCG GTC TC	50	822	[45]
*sul2*	F: CGG CAT CGT CAA CAT AAC C R: GTG TGC GGA TGA AGT CAG	50	722	[45]
*sul3*	F: CAA CGG AAG TGG GCG TTG TGG A R: GCT GCA CCA ATT CGC TGA ACG	66	244	[46]
*bla_TEM_*	F: GAG TAT TCA ACA TTT TCG T R: ACC AAT GCT TAA TCA GTG A	50	857	[45]
*tet(A)*	F: GTG AAA CCC AAC ATA CCC C R: GAA GGC AAG CAG GAT GTA G	50	887	[45]
*tet(B)*	F: CCT TAT CAT GCC AGT CTT GC R: ACT GCC GTT TTT TCG CC	50	773	[45]
*dhfrI*	F: AAG AAT GGA GTT ATC GGG ATT G R: GGG TAA AAA CTG GCC TAA AAT TG	50	391	[45]
*qnr*	F: GGG TAT GGA TAT TAT TGA TAA AG R: CTA ATC CGG CAG CAC TAT TTA	50	670	[47]
*aac(3)-IV*	F: CTT CAG GAT GGC AAG TTG GT R: TCA TCT CGT TCT CCG CTC AT	55	286	[47]
*cmlA*	F: CCG CCA CGG TGT TGT TGT TAT C R: CAC CTT GCC TGC CCA TCA TTA G	55	698	[47]
*catI*	F: AGT TGC TCA ATG TAC CTA TAA CC R: TTG TAA TTC ATT AAG CAT TCT GCC	55	547	[47]
*aac(3)-II*	F: ATA TCG CGA TGC ATA CGC GG R: GAC GGC CTC TAA CCG GAA GG	56	877	[48]
*aadA*	F: GTG GAT GGC GGC CTG AAG CC R: AAT GCC CAG TCG GCA GCG	62	525	[46]
*strA/strB*	F: ATG GTG GAC CCT AAA ACT CT R: CGT CTA GGA TCG AGA CAA AG	62	893	[46]

**Table A2:** PCR primers used for detection of virulence genes.

Pathovar	Target Gene	Sequence (5′ → 3′) of Primers	Size of Amplification Product (bp)	Reference
EHEC, EPEC	*escV*	F: ATT CTG GCT CTC TTC TTC TTT ATG GCT G R: CGT CCC CTT TTA CAA ACT TCA TCG C	544	[49]
	*ent*	F: TGG GCT AAA AGA AGA CAC ACT G R: CAA GCA TCC TGA TTA TCT CAC C	629	[49]
	*eaeA*	F: GAC CCG GCA CAA GCA TAA GC R: CCA CCT GCA GCA ACA AGA GG	384	[50]
Typical EPEC	*bfpB*	F: GAC ACC TCA TTG CTG AAG TCG R: CCA GAA CAC CTC CGT TAT GC	910	[49]
EHEC	*stx1*	F: ATA AAT CGC CAT TCG TTG ACT AC R: AGA ACG CCC ACT GAG ATC ATC	180	[50]
	*stx2*	F: GGC ACT GTC TGA AAC TGC TCC R: TCG CCA GTT ATC TGA CAT TCT G	255	[50]
	*hlyA*	F: GCA TCA TCA AGC GTA CGT TCC R: AAT GAG CCA AGC TGG TTA AGC T	534	[50]
	*saa*	F: CGT GAT GAA CAG GCT ATT GC R: ATG GAC ATG CCT GTG GCA AC	119	[50]
EIEC	*ipaH*	F: GAA AAC CCT CCT GGT CCA TCA GG R: GCC GGT CAG CCA CCC TCT GAG AGT AC	437	[49]
	*invE*	F: CGA TAG ATG GCG AGA AAT TAT ATC CCG R: CGA TCA AGA ATC CCT AAC AGA AGA ATC AC	766	[49]
EAEC	*astA*	F: TGC CAT CAA CAC AGT ATA TCC G R: ACG GCT TTG TAG TCC TTC CAT	102	[49]
	*aggR*	F: ACG CAG AGT TGC CTG ATA AAG R: AAT ACA GAA TCG TCA GCA TCA GC	400	[49]
	*pic*	F: AGC CGT TTC CGC AGA AGC C R: AAA TGT CAG TGA ACC GAC GAT TGG	1,111	[49]
ETEC	*elt*	F: GAA CAG GAG GTT TCT GCG TTA GGT G R: CTT TCA ATG GCT TTT TTT TGG GAG TC	655	[49]
	*estIa*	F: CCT CTT TTA GYC AGA CAR CTG AAT CAS TTG R: CAG GCA GGA TTA CAA CAA AGT TCA CAG	157	[49]
	*estIb*	F: TGTCTTTTTCACCTTTCGCTC R: CGGTACAAGCAGGATTACAACAC	171	[49]
APEC	*ompT*	F: TCA TCC CGG AAG CCT CCC TCA CTA CTA T R: TAG CGT TTG CTG CAC TGG CTT CTG ATA C	496	[51]
	*iutA*	F: GGC TGG ACA TCA TGG GAA CTG G R: CGT CGG GAA CGG GTA GAA TCG	302	[51]
	*irp-2*	F: AAG GAT TCG CTG TTA CCG GAC R: AAC TCC TGA TAC AGG TGG C	413	[52]
	*iss*	F: ATC ACA TAG GAT TCT GCC G R: CAG CGG AGT ATA GAT GCC A	306	[52]
	*pap-C*	F: TGA TAT CAC GCA GTC AGT AGC R: CCG GCC ATA TTC ACA TAA	501	[52]
	*tsh*	F: ACT ATT CTC TGC AGG AAG TC R: CTT CCG ATG TTC TGA ACG T	824	[52]
	*cva/cvi*	F: TGG TAG AAT GTG CCA GAG CAA G R: GAG CTG TTT GTA GCG AAG CC	1181	[52]
	*iucD*	F: ACA AAA AGT TCT ATC GCT TCC R: CCT GAT CCA GAT GAT GCT C	714	[52]

**Table A3:** PCR schemes used to detect virulence genes in *E. coli*.

PCR Type	Detected Genes	Annealing Temperature (°C)	Reference
Multiplex I	*stx1*, *stx2*, *hylA*, *eaeA*, *saa*	65 (10 cycles) then 62 (20 cycles)	[50]
Multiplex II	*ecsV*, *ent*, *bfpB*, *invE*, *astA*, *aggR*, *pic*, *ipaH*, *elt*, *estIa*, *estIb*	62	[49]
Multiplex III	*ompT*, *iutA*	63	[51]
Multiplex IV	*tsh*, *pap-C*, *iss*, *irp-2*	57	[52]
Uniplex I	*cva*/*cvi*	58	[52]
Uniplex II	*iucD*	55	[52]
